# Latent profiles analysis and associated factors of digital health literacy among young and middle-aged adults with diabetes

**DOI:** 10.3389/fpubh.2026.1865183

**Published:** 2026-08-04

**Authors:** Jingwen Song, Di Zhou, Wenli Tao, Chunyan Li, Juan Yuan

**Affiliations:** 1 School of Nursing, Anhui University of Chinese Medicine, Hefei, China; 2 Laboratory of Geriatric Nursing and Health, Anhui University of Chinese Medicine, Hefei, China

**Keywords:** associated factors, diabetes, digital health literacy, latent profile analysis, young and middle-aged adults

## Abstract

**Objective:**

Digital health technologies are increasingly used in chronic disease management, but their effective use requires adequate digital health literacy (DHL). Because an overall DHL score may mask heterogeneity across its multiple dimensions, this study aimed to identify latent profiles of DHL among young and middle-aged adults with diabetes and to examine factors associated with profile membership, thereby informing individualized health education and stratified health promotion strategies for diabetes management.

**Methods:**

Guided by the health ecology model, a cross-sectional study was conducted among 504 hospitalized patients with diabetes aged 18–59 years from two tertiary hospitals in Anhui Province, China, between June 2025 and March 2026. Data were collected using a general information questionnaire, the Digital Health Literacy Instrument, the Diabetes Self-Management Questionnaire, the Technophobia Scale, and the Social Support Rating Scale. Latent profile analysis (LPA) was used to identify DHL profiles. LASSO regression was applied to screen candidate variables, and multinomial logistic regression was used to examine factors associated with profile membership.

**Results:**

The mean DHLI score was 2.02 ± 0.61. LPA identified four profiles: limited DHL profile, basic application-low navigation profile, operational strength-limited critical appraisal profile, and high-level comprehensive DHL profile. Multinomial logistic regression showed that age, educational level, residence, complications, mobile health tool use, daily internet use, online health information seeking, diversity of information sources, technophobia, and social support were significantly associated with DHL profile membership (*p* < 0.05).

**Conclusion:**

DHL among young and middle-aged adults with diabetes showed distinct profile characteristics in information access, appraisal, interaction, and privacy protection. These findings provide a basis for individualized health education and stratified health promotion strategies based on patients’ DHL profiles in diabetes management.

## 1 Introduction

As of June 2025, the number of internet users in China had reached 1.123 billion, with an internet penetration rate of 79.7% (1). The implementation of the “Internet + Healthcare” policy has supported the growth of mobile health services, remote follow-up, and the use of digital tools in healthcare delivery (2, 3). China has the largest adult diabetes population worldwide (4). Its complications remain a major cause of disability (5–7), creating a long-term burden for both the healthcare system and patients’ day-to-day disease management (8). Another concern is that diabetes is increasingly affecting younger people. Prevalence has continued to increase among young and middle-aged adults, making this population especially important in current prevention and management efforts (9). Yet awareness, treatment, and glycemic control of diabetes remain suboptimal (10). Compared with older adults, young and middle-aged patients with diabetes are often exposed to the combined pressures of career development and family responsibilities, as well as fast-paced and irregular lifestyles. These may make glycemic management more difficult and self-management more challenging (11–13). Therefore, improving self-management capacity among young and middle-aged adults with diabetes has important implications for glycemic control, complication prevention, and reducing the broader social burden.

Self-management is a core component of diabetes care. According to the *China Diabetes Prevention and Treatment Guidelines (2024)*, patients are required to engage in long-term blood glucose monitoring, dietary control, exercise management and medication adherence (14). The rapid development of digital technologies has improved access to healthcare services for people with diabetes (15). The World Health Organization’s *Global Strategy on Digital Health 2020–2025* further emphasizes the integration of digital technologies into health systems to expand service coverage and enhance accessibility and equity (16). Health literacy refers to an individual’s ability to obtain, understand, and use health information to make appropriate health decisions (17). With the rapid development of digital health technologies, the concept of electronic health literacy (eHL) was introduced to describe individuals’ ability to access, understand, and use online health information (18). Digital health literacy (DHL) further extends this concept by integrating health literacy with the digital competencies required in digital health environments. In the context of Web 2.0, where health information seeking has shifted from passive information retrieval to active user interaction and content generation, DHL encompasses competencies related to interaction with digital health platforms, information sharing, critical appraisal of online content, navigation of digital services, and privacy protection (19). Among these competencies, navigation skills and privacy protection reflect individuals’ ability to effectively and safely engage with complex digital health systems and online health communities. Previous studies have shown that in patients with diabetes, a higher level of DHL is associated with better self-care, greater self-efficacy, and lower diabetes-related distress (20).

Despite these advances, previous studies have mainly focused on the overall level of DHL and its influencing factors. Less attention has been paid to heterogeneity in cognitive and behavioral patterns within patient populations. Latent profile analysis (LPA) is a person-centered statistical approach used to classify individuals into distinct latent profiles based on response patterns across multiple continuous indicators, thereby capturing population heterogeneity that may not be identified using variable-centered approaches (21). However, studies on the latent profiles of DHL among young and middle-aged adults with diabetes remain limited. The health ecology model (HEM) highlights that health behaviors and health-related capabilities are the product of multilevel influences acting together, including factors at the individual, interpersonal, organizational, community, and policy levels (22, 23). This model provides an appropriate theoretical framework for systematically examining factors related to DHL. Therefore, guided by HEM, this study aimed to identify latent profiles of DHL among young and middle-aged adults with diabetes using latent profile analysis and to examine factors associated with profile membership. The findings may provide a reference for developing targeted interventions and health promotion strategies for this population.

## 2 Methods

### 2.1 Study design and participants

#### 2.1.1 Sample size calculation

This study employed a cross-sectional design and used convenience sampling to recruit hospitalized patients with diabetes from two hospitals in Anhui Province, China, between June 2025 and March 2026. The reporting of this study followed the STROBE guidelines for cross-sectional studies. The sample size was calculated using the formula for cross-sectional studies (24), $n=\frac{\;\;U_{1-\alpha /2}^{2}S^{2}}{d^{2}}$ , where *U*_1-α/2_ is the critical value of the standard normal distribution. With the significance level set at α = 0.05, *U*_1-α/2_ was 1.96. In this formula, *S* represents the estimated standard deviation and *d* denotes the allowable error. The Digital Health Literacy Instrument (DHLI) score was used as the primary outcome. Based on a pilot survey of 50 participants, the standard deviation was estimated as *S* = 0.67. The allowable error was set at 10% of the standard deviation, namely *d* = 0.067. The minimum required sample size was therefore estimated to be approximately 384. In addition, previous studies have suggested that a sample size of at least 300–500 is generally recommended for latent profile analysis (LPA) to ensure robust model estimation (21). Considering a potential invalid response rate of 20%, at least 480 questionnaires were required. To further improve model stability for latent profile analysis, 520 questionnaires were ultimately distributed.

#### 2.1.2 Participants

The inclusion criteria were as follows: (1) a diagnosis of diabetes according to the China Diabetes Prevention and Treatment Guidelines (2024 Edition) (14); (2) provision of informed consent and voluntary participation in the study; (3) age between 18 and 59 years; (4) clear consciousness, no cognitive impairment, and the ability to communicate normally; and (5) ownership of a smartphone.

The exclusion criteria were as follows: (1) development of acute or critical illness during hospitalization, accompanied by severe complications or organ failure; (2) inability to cooperate because of severe visual, hearing, or speech impairment; and (3) gestational diabetes mellitus.

### 2.2 Variables and instruments

#### 2.2.1 General information questionnaire

Guided by the HEM (22), factors potentially associated with DHL in patients with diabetes were classified into multiple levels. The innermost level comprised core individual characteristics, including sex, age, type of diabetes, disease duration, family history, technophobia, comorbidities, and number of complications. The second level represented individual lifestyle and behavioral factors, including daily internet use, online health information seeking (OHIS) behavior, diversity of information sources, use of mobile health tools, and diabetes self-management. The third level reflected social and community network factors, including social support. The fourth level covered living and working conditions, including educational level, employment status, place of residence, and income. The outermost level consisted of broader socioeconomic, cultural, and policy-related factors, which were considered background factors and are discussed in the interpretation of the findings.

Use of mobile health tools was defined as any use of smartphone-based diabetes applications, telemedicine services, continuous glucose monitoring devices, or wearable devices for diabetes-related purposes during the past 3 months. Diversity of information sources was assessed according to the number of health information channels reported by participants, including search engines, short-video platforms, social media, and hospital official accounts.

#### 2.2.2 Digital Health Literacy Instrument (DHLI)

Digital health literacy was assessed using the Chinese version of the Digital Health Literacy Instrument, originally developed by van der Vaart and Drossaert (19) and cross-culturally adapted by Zhao et al. (25). The instrument assesses a broad range of digital health literacy skills, including operational skills, information searching, evaluating reliability, determining relevance, online communication, adding self-generated content, and privacy protection. These domains capture both functional and interactive competencies required for engagement in digital health environments, extending beyond traditional eHealth literacy frameworks. In this study, DHLI was used to assess patients’ digital health literacy. Items are rated on a 5-point Likert scale and scored from 4 to 0, with response options ranging from “very easy” to “very difficult” or from “always” to “never,” depending on the item content. The total score was calculated as the sum of item scores divided by the number of items completed, with higher scores indicating higher DHL. The original scale reported a Cronbach’s *α* of 0.929, and the Cronbach’s α in this study was 0.964.

#### 2.2.3 Diabetes Self-Management Questionnaire (DSMQ)

Diabetes self-management was assessed using the Chinese version of the Diabetes Self-Management Questionnaire, originally developed by Schmitt et al. (26) and cross-culturally adapted by Li et al. (27). This instrument evaluates patients’ daily diabetes self-management. The Chinese DSMQ includes 16 items across five dimensions: dietary control, physician contact, blood glucose monitoring, medication adherence, and physical activity. Each item is rated on a 4-point Likert scale from 0 to 3, with response options ranging from “does not apply” to “applies very much.” Items 5, 7, and 10–16 are reverse-scored. The total DSMQ score was calculated as (sum of all item scores/48) × 10, ranging from 0 to 10, with higher scores indicating better diabetes self-management. The original scale reported a Cronbach’s *α* of 0.764, and the Cronbach’s α in this study was 0.803.

#### 2.2.4 Social Support Rating Scale (SSRS)

Social support was assessed using the Social Support Rating Scale, developed by Xiao (28). The scale includes 10 items across three dimensions: subjective support, objective support, and support utilization. Items 1–4 and 8–10 are single-choice items scored from 1 to 4. Item 5 consists of five sub-items, each scored from 1 to 4, and the summed score is included in the subjective support dimension. Items 6 and 7 are multiple-choice items, scored as 0 when no source of support is reported and according to the number of reported sources when support is available. Total scores range from 12 to 66, with higher scores indicating greater social support. The original scale reported a Cronbach’s *α* of 0.89, and the Cronbach’s α in this study was 0.773.

#### 2.2.5 Technophobia Scale (TS)

Technophobia was assessed using the Chinese version of the Technophobia Scale, originally developed by Khasawneh (29) and cross-culturally adapted by Sun et al. in 2022 (30). This instrument evaluates the level of anxiety and fear individuals experience when encountering and using new technologies. The Chinese version consists of 13 items across three dimensions: techno-anxiety, techno-fear, and privacy and security concerns. Each item is rated on a 5-point Likert scale, with response options ranging from “strongly disagree” to “strongly agree” and scores from 1 to 5. Total scores range from 13 to 65, with higher scores indicating higher levels of technophobia. The original scale reported a Cronbach’s *α* of 0.911, and the Cronbach’s α in this study was 0.946.

### 2.3 Ethical considerations

This study was conducted in accordance with the Declaration of Helsinki and was approved by the Ethics Committee of Anhui University of Chinese Medicine (Approval No. AHUCM-HSS-2025122). All participants were informed of the study purpose, the voluntary nature of participation, their right to refuse to answer any question or to withdraw at any time, as well as the potential risks and benefits of participation. Written informed consent was obtained from all participants.

### 2.4 Data collection

Data were collected by trained researchers in the relevant hospital departments. Before the survey, all participants were informed of the study purpose, questionnaire content, confidentiality principles, and the voluntary nature of participation, and written informed consent was obtained. The survey was conducted in a quiet ward environment to ensure a relatively undisturbed setting. During data collection, researchers provided standardized instructions on questionnaire completion, and participants completed the questionnaires independently. All questionnaires were collected immediately after completion and checked on site for completeness. If any items were missing, participants were invited to complete them at the time of collection if they were willing. Afterward, two researchers independently reviewed all questionnaires, and those with obvious patterned responses were considered invalid and excluded from the analysis.

### 2.5 Statistical analysis

Statistical analyses were performed using SPSS 27.0, Mplus 8.3, and R 4.4.1. Continuous variables were described as mean ± standard deviation or median (interquartile range), depending on their distribution, while categorical variables were presented as frequencies and percentages. After the optimal latent profiles were identified, differences across the identified latent profiles were examined using one-way analysis of variance or the Kruskal-Wallis H test for continuous variables, and the chi-square test or Fisher’s exact test for categorical variables.

Latent profile analysis (LPA) was conducted in Mplus 8.3 to identify subgroups of DHL. Model fit was evaluated using the Akaike Information Criterion (AIC), Bayesian Information Criterion (BIC), adjusted Bayesian Information Criterion (aBIC), entropy, the Lo–Mendell–Rubin adjusted likelihood ratio test (LMRT), and the bootstrap likelihood ratio test (BLRT). Lower AIC, BIC, and aBIC values indicate better model fit, whereas higher entropy indicates greater classification accuracy. Significant LMRT and BLRT results (*p* < 0.05) indicate that the k-class model fits the data better than the k-1 class model. The optimal model was selected based on a comprehensive evaluation of these indices.

Based on the HEM, all candidate variables were entered into LASSO regression in R for variable selection, followed by multinomial logistic regression to identify factors associated with latent profile membership of DHL among young and middle-aged adults with diabetes. A two-sided *p* < 0.05 was considered statistically significant for the final model.

## 3 Results

### 3.1 Participant characteristics

A total of 520 participants met the inclusion criteria and were invited to complete the survey. After excluding 16 questionnaires due to missing or patterned responses, 504 valid questionnaires were retained, yielding an effective response rate of 96.9%. Participant characteristics are summarized in Table 1. Among the 504 participants, 351 (69.6%) were male and 153 (30.4%) were female. 42 (8.3%) were aged 18–37 years, 94 (18.7%) were aged 38–47 years, and 368 (73.0%) were aged 48–59 years. A total of 269 participants (53.4%) had a junior or senior high school education. Most participants lived in urban areas (81.5%), and 283 (56.2%) reported a monthly income of <5,000 RMB. Regarding diabetes-related characteristics, 495 participants (98.2%) had type 2 diabetes, 201 (39.9%) had a disease duration of ≥10 years, and 330 (65.5%) had no complications.

**Table 1:** Univariate analysis across latent profiles.

Variable	Classification	Overall (*n* = 504)	C1 (*n* = 94)	C2 (*n* = 79)	C3 (*n* = 108)	C4 (*n* = 223)	Statistic	*P*
Gender	Male	351 (69.6)	46 (48.9)	50 (63.3)	88 (81.5)	167 (74.9)	30.633 ^a^	<0.001
	Female	153 (30.4)	48 (51.1)	29 (36.7)	20 (18.5)	56 (25.1)		
Age (years)	18–37	42 (8.3)	1 (1.1)	1 (1.3)	2 (1.9)	38 (17.0)		
	38–47	94 (18.7)	2 (2.1)	4 (5.1)	16 (14.8)	72 (32.3)	109.818 ^a^	<0.001
	48–59	368 (73.0)	91 (96.8)	74 (93.7)	90 (83.3)	113 (50.7)		
Education level	Primary school and below	70 (13.9)	44 (46.8)	15 (19.0)	10 (9.3)	1 (0.4)		
	Junior or Senior high school	269 (53.4)	48 (51.1)	55 (69.6)	73 (67.6)	93 (41.7)	203.155 ^a^	<0.001
	Bachelor’s degree or above	165 (32.7)	2 (2.1)	9 (11.4)	25 (23.1)	129 (57.8)		
Employment status	Employed	201 (39.9)	12 (12.8)	21 (26.6)	46 (42.6)	122 (54.7)		
	Self-employed	110 (21.8)	13 (13.8)	14 (17.7)	32 (29.6)	51 (22.9)	103.432 ^a^	<0.001
	Farmer	27 (5.4)	15 (16.0)	7 (8.9)	2 (1.9)	3 (1.3)		
	Retired or unemployed	166 (32.9)	54 (57.4)	37 (46.8)	28 (25.9)	47 (21.1)		
Residence	Urban	411 (81.5)	50 (53.2)	53 (67.1)	93 (86.1)	215 (96.4)		
	Rural	93 (18.5)	44 (46.8)	26 (32.9)	15 (13.9)	8 (3.6)	95.447 ^a^	<0.001
Income	<5,000 RMB	283 (56.2)	85 (90.4)	66 (83.5)	54 (50.0)	78 (35.0)	111.189 ^a^	<0.001
	≥5,000 RMB	221 (43.8)	9 (9.6)	13 (16.5)	54 (50.0)	145 (65.0)		
Type of diabetes	Type 1	9 (1.8)	2 (2.1)	2 (2.5)	3 (2.8)	(0.9)	2.578 ^b^	0.44
	Type 2	495 (98.2)	92 (97.9)	77 (97.5)	105 (97.2)	221 (99.1)		
Disease duration	<3 years	161 (31.9)	22 (23.4)	23 (29.1)	21 (19.4)	95 (42.6)		
	3–9 years	142 (28.2)	30 (31.9)	13 (16.5)	34 (31.5)	65 (29.1)	34.954 ^a^	<0.001
	≥10 years	201 (39.9)	42 (44.7)	43 (54.4)	53 (49.1)	63 (28.3)		
Family history	Yes	243 (48.2)	38 (40.4)	34 (43.0)	46 (42.6)	125 (56.1)	9.988 ^a^	0.019
	No	261 (51.8)	56 (59.6)	45 (57.0)	62 (57.4)	98 (43.9)		
Complications	0	330 (65.5)	53 (56.4)	44 (55.7)	64 (59.3)	169 (75.8)		
	1	103 (20.4)	23 (24.5)	16 (20.3)	27 (25.0)	37 (16.6)	24.16 ^a^	<0.001
	≥2	71 (14.1)	18 (19.1)	19 (24.1)	17 (15.7)	17 (7.6)		
Comorbid chronic diseases	0	235 (46.6)	36 (38.3)	32 (40.5)	60 (55.6)	107 (48.0)	17.509 ^a^	0.008
	1	154 (30.6)	24 (25.5)	27 (34.2)	27 (25.0)	76 (34.1)		
	≥2	115 (22.8)	34 (36.2)	20 (25.3)	21 (19.4)	40 (17.9)		
Daily internet use	<3 h	227 (45.0)	73 (77.7)	56 (70.9)	61 (56.5)	37 (16.6)		
	3–5 h	187 (37.1)	15 (16.0)	13 (16.5)	34 (31.5)	125 (56.1)	141.481 ^a^	<0.001
	>5 h	90 (17.9)	6 (6.4)	10 (12.7)	13 (12.0)	61 (27.4)		
OHIS	<1 time/week or never	249 (49.4)	85 (90.4)	60 (75.9)	55 (50.9)	49 (22.0)		
	Weekly	214 (42.5)	8 (8.5)	18 (22.8)	47 (43.5)	141 (63.2)	156.114 ^a^	<0.001
	Daily	41 (8.1)	1 (1.1)	1 (1.3)	6 (5.6)	33 (14.8)		
Information source diversity	1	190 (37.7)	82 (87.2)	34 (43.0)	29 (26.9)	45 (20.2)	133.716 ^a^	<0.001
	≥2	314	(62.3)	12 (12.8)	45 (57.0)	79 (73.1)	178 (79.8)	
Mobile health tools use	Yes	84 (16.7)	1 (1.1)	2 (2.5)	13 (12.0)	68 (30.5)	60.203 ^a^	<0.001
	No	420 (83.3)	93 (98.9)	77 (97.5)	95 (88.0)	155 (69.5)		
DSMQ score, mean ± SD	-	6.25 ± 1.04	5.79 ± 1.14	6.00 ± 1.01	6.33 ± 0.97	6.49 ± 0.96	12.47 ^c^	<0.001
SSRS score, mean ± SD	-	35.46 ± 8.07	27.85 ± 6.22	30.84 ± 6.00	35.24 ± 6.49	40.42 ± 6.44	104.6 ^c^	<0.001
TS score, mean ± SD	-	28.00 (6.00)	32.50 (13.00)	28.00 (6.00)	26.00 (5.00)	23.00 (10.00)	143.01 ^d^	<0.001

DSMQ, Diabetes Self-Management Questionnaire; SSRS, Social Support Rating Scale; TS, Technical Anxiety Scale; OHIS, online health information seeking. Data are presented as n (%), mean ± SD, or median (IQR), as appropriate.

a Chi-square test.

b Fisher’s exact test.

c One-way analysis of variance.

d Kruskal–Wallis H test.

### 3.2 Latent profile analysis of digital health literacy

Using the seven dimensions of the DHLI as observed indicators, latent profile analysis was conducted to identify latent profiles of DHL among young and middle-aged adults with diabetes. Models with 1 to 5 latent profiles were fitted sequentially, and the results are presented in Table 2. As the number of classes increased, the AIC, BIC, and aBIC generally decreased, although the magnitude of decline became less pronounced after the 3-class model. Compared with the 2-class model, the 3-class model showed lower AIC, BIC, and aBIC values, and both the LMRT and BLRT were statistically significant (*p* < 0.001), indicating an improved model fit.

**Table 2:** Model fit indices for latent profile analysis of digital health literacy.

Model	AIC	BIC	aBIC	Entropy	*P* = value		Class proportion (%)
					LMRT	BLRT	
Class 1	7635.888	7695.004	7650.567	—	—	—	—
Class 2	5393.896	5486.793	5416.963	0.957	<0.001	<0.001	34.9/65.1
Class 3	4792.800	4919.478	4824.255	0.956	<0.001	<0.001	23.2/30.2/46.6
Class 4	4546.848	4707.306	4586.690	0.946	0.0106	<0.001	18.7/15.7/21.4/44.2
Class 5	4333.412	4527.650	4381.642	0.933	0.2312	<0.001	14.3/9.3/17.5/18.1/40.9

AIC, Akaike information criterion; BIC, Bayesian information criterion; aBIC, sample-size adjusted Bayesian information criterion; LMRT, Lo–Mendell–Rubin likelihood ratio test; BLRT, bootstrap likelihood ratio test.

Further comparison between the 3-class and 4-class models showed that the 4-class model yielded additional reductions in AIC, BIC, and aBIC, while the LMRT and BLRT remained statistically significant. The entropy value for the 4-class model was 0.946. Considering class distribution and interpretability, all four latent profiles had relatively balanced sample sizes, with the smallest and largest classes representing 15.7 and 44.2% of the sample, respectively, which supports stable estimation and meaningful clinical interpretation. Although the AIC, BIC, and aBIC continued to decrease in the five-class model, the LMRT was not statistically significant (*p* = 0.231), suggesting that the addition of a fifth class did not significantly improve model fit over the four-class solution. Moreover, the fifth class accounted for only 9.3% of the sample, which may reduce model stability and limit clinical interpretability. Therefore, the four-class model was selected as the optimal solution by balancing statistical fit, parsimony, stability, and clinical interpretability.

### 3.3 Profile labeling of digital health literacy

#### 3.3.1 Average latent class probabilities

The reliability of the latent profile assignment was assessed using average posterior probabilities. The diagonal values for the four latent profiles ranged from 0.927 to 0.990, suggesting that most participants were classified into their respective profiles with a relatively high degree of certainty. Off-diagonal probabilities were generally low, indicating limited classification ambiguity. These findings indicate that the latent profiles are reasonably well-separated and that the classification can be considered reliable. Detailed probabilities for each profile are presented in Table 3.

**Table 3:** Average posterior probabilities for the final 4-class model.

Class	C1	C2	C3	C4
C1	0.985	0.015	0.000	0.000
C2	0.011	0.960	0.029	0.000
C3	0.000	0.048	0.927	0.024
C4	0.000	0.000	0.010	0.990

#### 3.3.2 Profile characteristics and naming

**Figure 1:**
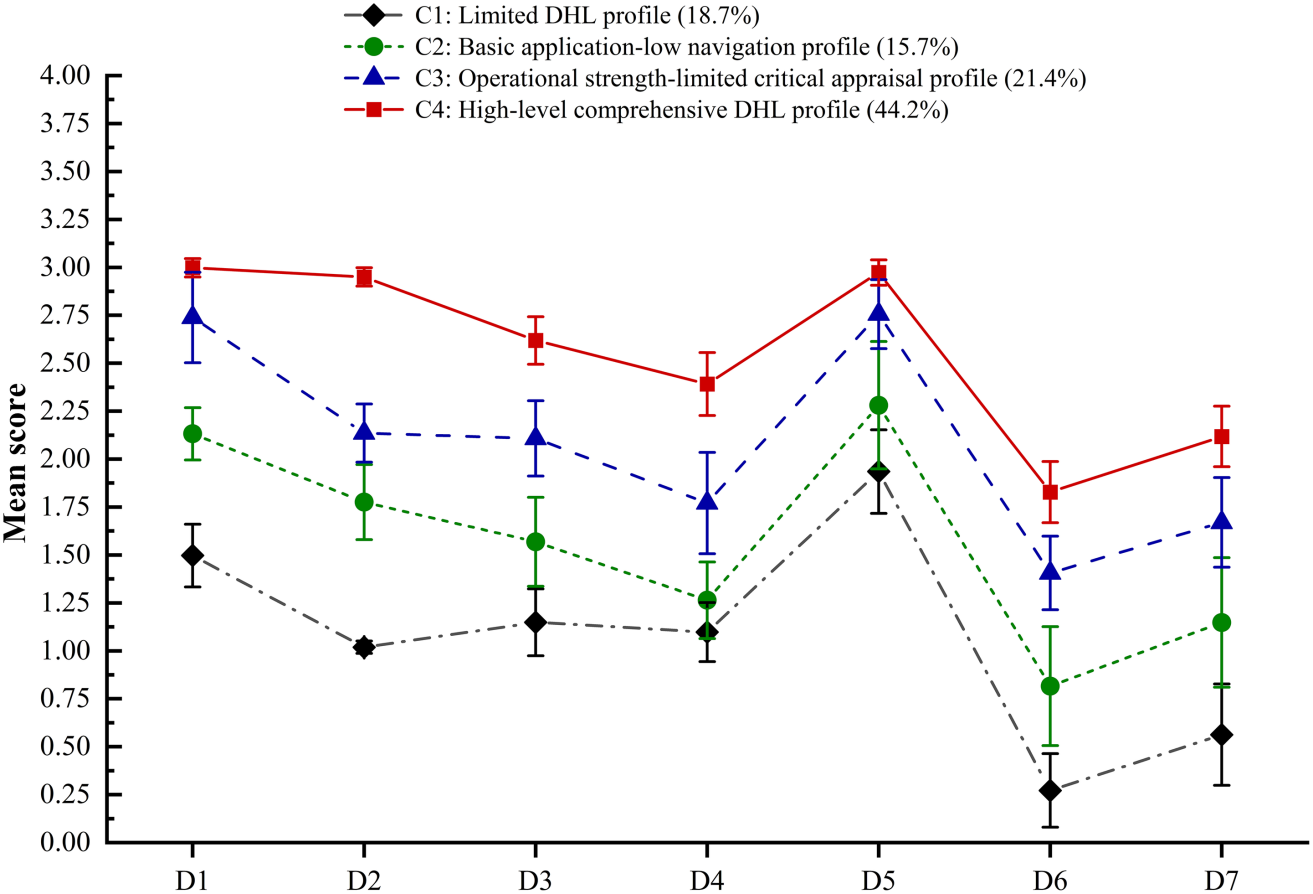
Distribution of the four latent profiles of digital health literacy among young and middle-aged adults with diabetes. D1, operational skills; D2, information searching; D3, determining relevance; D4, evaluating reliability; D5, adding content; D6, navigation skills; D7, protecting privacy. C1, limited DHL profile; C2, basic application-low navigation profile; C3, operational strength-limited critical appraisal profile; C4, high-level comprehensive DHL profile. Error bars represent 95% confidence intervals for the mean score of each profile on each dimension.

The four latent profiles were identified and named based on their conditional mean patterns across the seven DHLI dimensions (Figure 1). DHL scores across latent profiles are shown in Table 4.

**Table 4:** Digital health literacy scores across latent profiles.

Variables	Mean DHLI score	C1	C2	C3	C4
Operational skills	2.52 ± 0.63	1.50 ± 0.34	2.11 ± 0.25	2.74 ± 0.35	3.00 ± 0.16
Information searching	2.23 ± 0.76	1.02 ± 0.08	1.78 ± 0.26	2.13 ± 0.24	2.95 ± 0.16
Determining relevance	2.07 ± 0.71	1.16 ± 0.36	1.56 ± 0.46	2.10 ± 0.41	2.62 ± 0.44
Evaluating reliability	1.84 ± 0.75	1.10 ± 0.35	1.26 ± 0.41	1.76 ± 0.59	2.40 ± 0.58
Adding content	2.62 ± 0.55	1.94 ± 0.47	2.27 ± 0.46	2.75 ± 0.41	2.98 ± 0.23
Navigation skills	1.29 ± 0.79	0.28 ± 0.42	0.79 ± 0.52	1.41 ± 0.47	1.83 ± 0.57
Protecting privacy	1.58 ± 0.83	0.56 ± 0.59	1.13 ± 0.58	1.69 ± 0.57	2.11 ± 0.59
DHL total	2.02 ± 0.61	1.08 ± 0.23	1.56 ± 0.18	2.08 ± 0.21	2.56 ± 0.24

Values are presented as mean ± SD.

Class 1 was named the limited DHL profile. This class accounted for 18.7% of the sample and had the lowest scores across all seven dimensions, with a mean DHLI score of 1.08 ± 0.23. Patients in this class showed weak performance throughout the main process of digital health information use. They had difficulties not only in using digital devices and searching for health information, but also in selecting relevant information, judging information reliability, expressing health concerns online, seeking help through digital channels, and protecting personal privacy.

Class 2 was named the basic application–low navigation profile. This class represented 21.4% of the sample. Patients in this class had some basic ability in operational skills and adding content, with higher scores than Class 1 in these two dimensions. They were able to perform simple digital tasks and express health-related questions to some extent. However, their performance was weaker in information searching, determining relevance, evaluating reliability, and especially in navigation skills. They had more difficulty using appropriate search paths, locating useful health resources, and seeking help through online platforms or discussions. The main feature of this class was basic digital application ability combined with poor navigation ability.

Class 3 was named the operational strength–limited critical appraisal profile. This class accounted for 15.7% of the sample. Patients in this class had relatively high scores in operational skills and adding content, close to those of Class 4. They were able to use mobile health tools and express health concerns clearly. However, their scores in evaluating reliability, navigation skills, and protecting privacy were lower than those of the high-level profile. Compared with their stronger operational performance, they were less able to evaluate the reliability of online health information, identify commercial information, compare information from different websites, locate useful resources, and protect privacy in digital environments.

Class 4 was named the high-level comprehensive DHL profile. This was the largest class, accounting for 44.2% of the sample. Patients in this class had the highest scores across all seven dimensions, with a mean DHLI score of 2.56 ± 0.24. They performed well in using digital devices, searching for health information, judging relevance, evaluating reliability, expressing health concerns online, navigating digital resources, and protecting privacy. Although navigation skills remained the relatively lowest dimension within this class, their overall performance was consistently higher than that of the other three classes.

### 3.4 Univariate analysis of digital health literacy across latent profiles

The univariate analysis showed significant differences across the 4 latent profiles in sex, age, educational level, employment status, place of residence, monthly household income, disease duration, family history, number of complications, number of chronic comorbidities, daily internet use, frequency of OHIS, diversity of information sources, use of mobile health tools, DSMQ score, SSRS score, and TS score (*p* < 0.05), as shown in Table 1.

### 3.5 Variable selection

**Figure 2:**
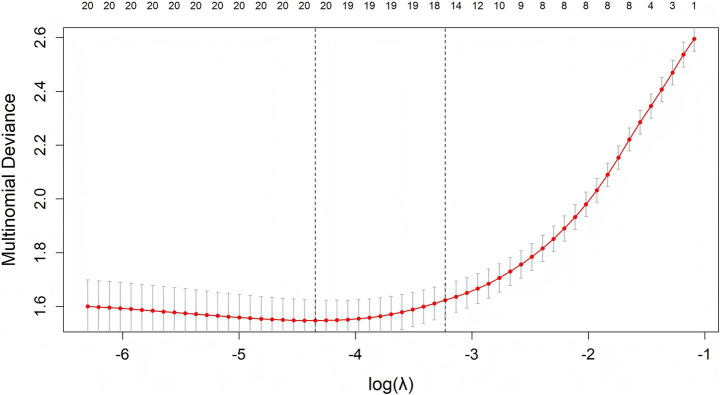
Selection of the optimal tuning parameter (*λ*) in the LASSO regression model. The *y*-axis represents the multinomial deviance, and the *x*-axis shows log(*λ*) values. Each point indicates the mean cross-validated deviance with corresponding standard error bars. The two vertical dashed lines represent *λ*_min_ (the value of *λ* that minimizes cross-validated deviance) and *λ*_1se_ (the most regularized model within one standard error of the minimum). The numbers at the top indicate the number of variables retained in the model at each *λ* value.

Based on the HEM, 18 candidate variables were entered into a LASSO-penalized multinomial logistic regression to select those associated with DHL latent profile membership. The penalty parameter was determined using 10-fold cross-validation with the glmnet package in R. The results showed that the penalty parameter corresponding to the minimum cross-validation error was lambda.min = 0.00956, and the penalty parameter within 1 standard error of the minimum was lambda.1se = 0.0242. Based on the principle of model parsimony, lambda.1se was ultimately selected in this study. A total of 15 variables were retained, including age, gender, educational level, employment status, place of residence, income, disease duration, complications, chronic comorbidities, use of mobile health tools, daily internet use, OHIS, diversity of information sources, technophobia, and social support. Multicollinearity among these 15 variables was assessed using variance inflation factors (VIF). All VIF values were below 5 (mean VIF = 1.87), indicating no severe multicollinearity. Figure 2 shows the 10-fold cross-validation error curve for the LASSO model, and the numbers at the top indicate the number of nonzero coefficients at each value of *λ*.

### 3.6 Multivariable analysis of latent DHL profiles

A multinomial logistic regression model was fitted using the multinom function in the nnet package in R. The 15 variables selected by the LASSO regression were entered into the model (variable coding is shown in Table 5), with the high-level comprehensive DHL profile as the reference profile. The results showed that age, educational level, place of residence, complications, use of mobile health tools, daily internet use, OHIS, diversity of information sources, technophobia, and social support were independent factors associated with latent profile membership of DHL among young and middle-aged adults with diabetes (*p* < 0.05), as shown in Table 6. As a sensitivity analysis, we entered all variables from the health ecology model (HEM) into a multinomial logistic regression model. Complete results are shown in Supplementary Table S1.

**Table 5:** Variable coding.

Variables	Assignment method
Age	1 = 18–37 years; 2 = 38–47 years; 3 = 48–59 years
Gender	1 = Male; 2 = Female
Education level	1 = Primary or below; 2 = Junior/Senior high school; 3 = Bachelor’s degree or above
Employment status	1 = Government/enterprise employee; 2 = Self-employed; 3 = Farmer; 4 = Retired/unemployed
Residence	1 = Urban; 2 = Rural
Income	1 = <5,000 RMB; 2 = ≥5,000 RMB
Disease duration	1 = <3 years; 2 = 3–9 years; 3 = ≥10 years
Complications	1 = 0; 2 = 1; 3 = ≥2
Comorbid chronic diseases	1 = 0; 2 = 1; 3 = ≥2
Daily internet use	1 = <3 h; 2 = 3–5 h; 3 = > 5 h
OHIS	1 = <1 time/week; 2 = Weekly; 3 = Daily
Information source diversity	1 = Single type; 2 = ≥2 types
Mobile health tools use	1 = Yes; 2 = No
Technophobia	Z-score
Social support	Z-score

**Table 6:** Multinomial logistic regression of factors associated with latent profiles.

Variables	C1 vs. C4			C2 vs. C4			C3 vs. C4		
	β	OR (95% CI)	*P*	β	OR (95% CI)	*P*	β	OR (95% CI)	*P*
Gender (ref: Male)									
Female	−0.205	0.815 (0.231–2.877)	0.75	−0.058	0.944 (0.324–2.751)	0.916	−0.337	0.714 (0.304–1.676)	0.439
Age (ref: 18–37 years)									
38–47 years	0.519	1.680 (0.023–120.600)	0.812	1.036	2.818 (0.141–56.230)	0.498	0.942	2.565 (0.410–16.060)	0.314
48–59 years	3.959	52.380 (1.197–2293.100)	0.040	3.140	23.110 (1.443–370.300)	0.026	2.301	9.986 (1.717–58.080)	0.010
Education level (ref: primary school or below)									
Junior/senior high school	−2.859	0.057 (0.006–0.594)	0.017	−1.686	0.185 (0.019–1.813)	0.147	−1.458	0.233 (0.025–2.159)	0.200
Bachelor’s degree or above	−5.28	0.005 (0.000–0.099)	<0.001	−2.92	0.054 (0.004–0.669)	0.023	−2.779	0.062 (0.006–0.631)	0.019
Employment status (ref: employee)									
Self-employed	−0.692	0.501 (0.109–2.308)	0.375	−0.417	0.659 (0.192–2.262)	0.507	−0.008	0.992 (0.422–2.330)	0.985
Farmer	−1.246	0.288 (0.013–6.331)	0.430	−1.430	0.239 (0.014–3.993)	0.319	−1.908	0.148 (0.010–2.169)	0.163
Retired or unemployed	0.210	1.233 (0.295–5.161)	0.774	−0.196	0.822 (0.266–2.542)	0.734	−0.432	0.649 (0.268–1.576)	0.340
Residence (ref: Urban)									
Rural	1.491	4.440 (0.736–26.800)	0.104	1.736	5.676 (1.066–30.230)	0.042	1.531	4.622 (1.070–19.960)	0.040
Income (ref: <5,000 RMB)									
≥5,000 RMB	−0.313	0.731 (0.160–3.337)	0.686	−0.480	0.619 (0.196–1.951)	0.413	0.585	1.796 (0.820–3.934)	0.144
Disease duration (ref: <3 years)									
3–9 years	0.278	1.321 (0.318–5.489)	0.702	−1.039	0.354 (0.109–1.148)	0.084	0.365	1.440 (0.612–3.390)	0.403
≥10 years	0.042	1.043 (0.262–4.158)	0.952	−0.373	0.688 (0.231–2.049)	0.502	0.316	1.372 (0.581–3.239)	0.471
Complications (ref: 0)									
1	0.646	1.907 (0.518–7.017)	0.331	0.490	1.633 (0.541–4.931)	0.384	0.438	1.550 (0.675–3.556)	0.301
≥2	0.629	1.875 (0.383–9.176)	0.438	1.383	3.985 (1.092–14.550)	0.036	0.876	2.401 (0.847–6.806)	0.100
Comorbid chronic diseases (ref: 0)									
1	−0.082	0.921 (0.281–3.013)	0.892	0.138	1.147 (0.437–3.010)	0.780	−0.644	0.525 (0.252–1.095)	0.086
≥2	1.141	3.130 (0.847–11.570)	0.087	0.564	1.757 (0.572–5.399)	0.325	−0.387	0.679 (0.291–1.584)	0.370
Mobile health tools use (ref: Yes)									
No	3.179	24.030 (1.374–420.200)	0.029	2.186	8.901 (1.290–61.410)	0.027	0.477	1.612 (0.652–3.988)	0.302
Daily internet use (ref: <3 h)									
3–5 h	−1.868	0.154 (0.045–0.531)	0.003	−2.037	0.130 (0.048–0.356)	<0.001	−1.312	0.269 (0.132–0.551)	<0.001
>5 h	−3.459	0.032 (0.006–0.169)	<0.001	−2.15	0.116 (0.032–0.423)	0.001	−1.57	0.208 (0.084–0.515)	0.001
OHIS (ref: <1 time/week or never)									
Weekly	−2.993	0.050 (0.013–0.195)	<0.001	−2.171	0.114 (0.043–0.306)	<0.001	−1.162	0.313 (0.150–0.651)	0.002
Daily	−4.384	0.013 (0.000–0.377)	0.012	−4.644	0.010 (0.001–0.169)	0.001	−1.385	0.250 (0.071–0.879)	0.031
Information source diversity (ref: single type)									
≥2 types	−2.587	0.075 (0.023–0.246)	<0.001	−0.565	0.569 (0.216–1.496)	0.253	−0.337	0.714 (0.324–1.576)	0.405
Technophobia	0.421	1.524 (1.046–2.219)	0.028	1.192	3.295 (1.911–5.681)	<0.001	1.837	6.273 (3.198–12.305)	<0.001
Social support	−0.797	0.451 (0.292–0.694)	<0.001	−1.210	0.298 (0.169–0.526)	<0.001	−1.479	0.228 (0.117–0.445)	<0.001

β, regression coefficient; OR, Odds Ratio; CI, confidence interval. C1 = limited DHL profile; C2 = basic application–low navigation profile; C3 = operational strength–limited critical appraisal profile; C4 = high-level comprehensive DHL profile.

### 3.7 Pairwise effect sizes for self-management, technology anxiety, and social support across DHL profiles

Significant differences were observed among the latent DHL profiles across the main outcome measures. For diabetes self-management, C4 showed the highest scores, whereas C1 showed the lowest scores. The largest difference was observed between C1 and C4 (d = −0.683), while moderate effects were found for C1 vs. C3 (d = −0.511) and C2 vs. C4 (d = −0.502). In contrast, technology anxiety was highest in C1 and lowest in C4, with large effects observed for C1 vs. C4 (d = 1.463), C1 vs. C3 (d = 1.193), and C2 vs. C4 (d = 1.003). For social support, C4 consistently showed the highest scores, with notable effect sizes observed for C1 vs. C4 (d = −1.973), C2 vs. C4 (d = −1.515), and C1 vs. C3 (d = −1.161) (Table 7).

**Table 7:** Pairwise comparisons of diabetes self-management, social support, and technical anxiety among the four DHL profiles.

Variables	C1 vs. C2	C1 vs. C3	C1 vs. C4	C2 vs. C3	C2 vs. C4	C3 vs. C4
Diabetes Self-Management	−0.190 (−0.489, 0.108)	−0.511 (−0.783, −0.239)	−0.683 (−0.918, −0.448)	−0.338 (−0.626, −0.049)	−0.502 (−0.756, −0.249)	−0.160 (−0.389, 0.070)
Technophobia	0.575 (0.270, 0.880)	1.193 (0.893, 1.493)	1.463 (1.197, 1.730)	0.674 (0.375, 0.972)	1.003 (0.734, 1.271)	0.466 (0.234, 0.699)
Social support	−0.488 (−0.782, −0.193)	−1.161 (−1.413, −0.908)	−1.973 (−2.158, −1.787)	−0.700 (−0.982, −0.419)	−1.515 (−1.742, −1.289)	−0.803 (−1.024, −0.581)

C1 = limited DHL profile; C2 = basic application–low navigation profile; C3 = operational strength–limited critical appraisal profile; C4 = high-level comprehensive DHL profile. The effect size is Cohen’s d, and the parentheses contain the 95% confidence interval.

## 4 Discussion

### 4.1 Status of digital health literacy and self-management in young and middle-aged adults with diabetes

In this study, young and middle-aged adults with diabetes had a mean DHLI score of 2.02 ± 0.61, indicating a moderate level of DHL. This value was lower than the 2.69 ± 0.61 reported by Zhao et al. (31) among Chinese internet users. Compared with findings from other countries, it was also lower than the baseline DHLI score of 2.97 ± 0.60 reported by van Schalkwijk et al. (32) in cardiovascular outpatients and the mean score of 3.04 ± 0.50 reported by Çetin and Gümüş (33) among Turkish adults aged 18–64 years from different generations. From the perspective of the HEM, this finding may reflect the combined influence of individual disease-management demands and the broader external support environment. At the individual level, diabetes, as a chronic condition requiring long-term self-management, places greater demands on patients’ ability to identify, evaluate, and apply digital health information. At the same time, DHL has been shown to vary across countries and regions, and these differences may be related to factors such as educational policy, cultural background, and economic conditions (34).

In the present study, young and middle-aged adults with diabetes had a mean self-management score of 6.25 ± 1.04, which was slightly higher than the level reported by Gu et al. (35). A recent systematic review and meta-analysis by Magi et al. (36), encompassing 138 studies with 52 included in the meta-analysis, reported a moderate positive association between health literacy and self-care behaviors in patients with chronic diseases. Previous studies in patients with diabetes have also reported a significant positive association between DHL and diabetes self-management (37). While traditional health literacy provides the foundation for self-care, DHL may represent a more digitally oriented pathway for supporting diabetes self-management. Individuals with higher DHL may be more capable of navigating digital health platforms, identifying reliable online information, communicating with healthcare providers, and applying digital resources to daily diabetes care. These abilities may support medication adherence, blood glucose monitoring, dietary management, and other self-management behaviors.

### 4.2 Latent profile characteristics of digital health literacy among young and middle-aged adults with diabetes

Profile C1 was labelled as the limited DHL profile, accounting for 18.7% of the sample. These patients had the lowest scores across all seven DHLI dimensions, with a mean DHLI score of 1.08 ± 0.23. This suggests broad difficulties in using digital health resources. Patients in Profile C1 had limited ability to express health concerns online and faced clear barriers in using basic device functions, searching for health information, and selecting relevant information. Their relatively lower educational attainment, income, and social support may further restrict access to and effective use of digital health resources. For patients in Profile C1, interventions could provide stepwise digital health support, beginning with guided training on basic device operation and simple online tasks, followed by gradual introduction of health information searching and evaluation skills. Information should be presented in a modular and visually accessible format, complemented by real-time support from family members or healthcare providers to enhance patient confidence and engagement.

Profile C2 was labelled as the basic application-low navigation profile, accounting for 21.4% of the sample. These patients had moderate scores for operational skills (2.11 ± 0.25) and adding content (2.27 ± 0.46); they were able to complete basic tasks on smartphones or other mobile devices. However, patients in Profile C2 remained relatively weak in determining relevance (1.56 ± 0.46) and evaluating reliability (1.26 ± 0.41). The most prominent characteristic of patients is their low score for navigation skills (0.79 ± 0.52), which is higher than that of only Profile C1 among the four subgroups. Although patients in profile C2 had access to relatively diverse information sources, they searched for online health information less often. Their use of digital health resources may remain passive and fragmented. For patients in Profile C2, interventions could focus on improving digital navigation by simplifying mobile health application pathways, providing stepwise visual guides, or incorporating voice-assisted search functions to facilitate more efficient use of health resources.

Profile C3 was labelled as the operational strength–limited critical appraisal profile, accounting for 15.7% of the sample. Patients in Profile C3 had relatively high scores for operational skills and adding content, close to those of Profile C4. They were able to use digital tools and express health concerns clearly. However, patients in Profile C3 still had relatively low scores for evaluating reliability (1.76 ± 0.59) and navigation skills (1.41 ± 0.47), which shows that being able to use digital tools does not necessarily mean being able to judge the quality of health information. Most participants in Profile C3 were aged 48–59 years, had a junior or senior high school education, and had a longer disease duration. Social support levels and use of mobile health tools were relatively low. For patients in Profile C3, training in critical appraisal and information evaluation should be provided to guide them in identifying reliable information sources and distinguishing advertisements and commercial content. Additionally, navigation and information retrieval skills should be developed to enable the safe and effective use of digital health tools in daily health management.

Profile C4 was labelled as the high-level comprehensive DHL profile, accounting for 44.2% of the sample and represented the largest profile. Patients in Profile C4 had relatively high scores across all DHLI dimensions, with a mean DHLI score of 2.56 ± 0.24. Most participants had a university education, lived in urban areas, reported stronger social support, and had better self-management of diabetes. Patients in Profile C4 were generally able to use digital tools, search for health information, evaluate information reliability, communicate online, identify risks, and protect privacy. However, navigation skills (1.83 ± 0.57) remained the weakest dimension even in this profile. Even among patients with relatively high DHL, there remained room for improvement in proactively seeking help and accurately identifying relevant resources. For patients in Profile C4, navigation skills and advanced search strategies should be further strengthened, and patients should be encouraged to actively seek professional health resources while integrating information retrieval with self-management practices.

### 4.3 Factors associated with digital health literacy profiles among young and middle-aged adults with diabetes

#### 4.3.1 Individual-level characteristics

Compared with the high-level comprehensive DHL profile (C4), patients aged 45–60 years were significantly more likely to be classified into the limited (C1), basic application–low navigation (C2), and operational strength–limited critical appraisal (C3) profiles. Previous studies have shown that age is an independent factor associated with DHL, with DHL tending to decline as age increases (38). From the perspective of the individual-level domain of the HEM, younger patients may have more frequent exposure to digital technologies, which may facilitate familiarity with smartphones and other digital devices. By contrast, age-related declines in cognitive function and sensory abilities can affect the learning, understanding, and use of online health information (39, 40). In practice, digital health interventions should pay greater attention to patients approaching older adulthood, and training strategies should be tailored to the characteristics of different age profiles.

In addition, higher levels of technophobia were associated with lower odds of belonging to the high-level comprehensive digital health literacy profile (C4), compared with all other profiles. According to the Technology Acceptance Model, technology acceptance is largely influenced by perceived usefulness and perceived ease of use (41). In the context of digital health technology adoption, technophobia has been regarded as an important psychological barrier to perceived ease of use. Higher levels of technophobia can increase patients’ perceived difficulty in operating digital health tools, reduce perceived ease of use, and thereby weaken their willingness to engage with such technologies (42). Therefore, clinical practice should also address patients’ psychological resistance and confidence in technology use, so as to reduce frustration in digital environments.

This study also found that patients with multiple complications were more likely to be classified in the basic application–low navigation profile (C2) than in the high-level comprehensive DHL profile (C4). Previous research has reported that patients with lower health literacy often experience poorer disease control and a greater burden of complications (43). According to the cumulative complexity model, health outcomes in patients with chronic disease depend on the dynamic balance between illness-related workload and individual capacity (44). The presence of multiple complications may increase the demands of medication management, symptom monitoring, lifestyle adjustment, and healthcare communication. This greater illness-related workload may occupy patients’ cognitive, emotional, and time resources, potentially limiting their capacity to develop digital skills and engage in active online health information seeking (45, 46). For patients with multiple complications, healthcare providers may consider reducing the burden of digital self-management by simplifying platform design, presenting health information more clearly, and providing individualized guidance with continuous follow-up support.

#### 4.3.2 Living and working conditions level

This study showed that patients living in rural areas were more likely to belong to the basic application–low navigation profile (C2) and the operational strength–limited critical appraisal profile (C3). Previous studies have indicated that place of residence is an important factor associated with DHL, with urban older adults generally reporting higher levels than their rural counterparts (47). In recent years, internet access in rural China has continued to improve. By June 2025, the rural internet penetration rate had reached 69.2% (1). However, improved access does not necessarily translate into better use-related skills or greater health benefits. From the perspective of digital divide theory, disparities among rural patients were no longer limited to the first-level access divide, but increasingly reflected the second-level divide in use-related skills and the third-level divide in benefits gained from digital technologies (48). Policymakers could place greater emphasis on strengthening infrastructure in rural areas and improving the accessibility of digital health services for disadvantaged profiles as digital health continues to advance.

In addition, patients with higher educational attainment were more likely to belong to the high-level comprehensive DHL profile. This finding is consistent with previous studies (49). Higher education is generally associated with stronger abilities to access, understand, and integrate information, and can also help individuals compare, evaluate, and apply information more effectively in complex information environments (50). In nursing practice and health education, more attention should be given to patients with lower educational attainment. Plain language, visual materials, and step-by-step instructions can help reduce the learning demands of digital health tools and avoid widening health inequalities through digital interventions.

#### 4.3.3 Behavioral characteristics level

Patients with longer daily internet use and more frequent OHIS were more likely to belong to the high-level comprehensive DHL profile. This finding is consistent with that of Liu et al. (51), who suggested that OHIS is not only an information acquisition behavior but also an important practical process in the development of DHL. From the perspective of the HEM, the behavioral characteristics level links individuals with the external digital environment. Frequent internet exposure can increase familiarity with different platform interfaces and help strengthen internet use and information-searching skills (52). Lee and Shim also found that middle-aged adults with diabetes who held more positive attitudes toward online health information had higher levels of DHL (53).

At the same time, compared with the high-level comprehensive DHL profile (C4), patients with more diverse information sources were significantly less likely to be classified into the limited DHL profile (C1). Previous research has likewise shown that the number of online health information channels is an important factor associated with DHL (54). Accessing health information through multiple channels can help young and middle-aged adults with diabetes encounter a broader range of information related to diet, exercise, and treatment. By contrast, reliance on a single platform can increase exposure to personalized recommendation algorithms and contribute to narrowed information exposure or even an “information cocoon” (55). In clinical practice, patients should be encouraged to seek health information online, while also being guided to develop a multi-source, comparable, and verifiable approach to health information acquisition.

Compared with patients in the high-level comprehensive DHL profile (C4), those who did not use mobile health tools were more likely to be classified into the limited (C1) and basic application–low navigation (C2) profiles. This finding is consistent with previous research showing that users of wearable devices generally demonstrate higher levels of DHL (56). Tools such as continuous glucose monitoring (CGM) require users to interpret data, identify trends, and adjust management behaviors accordingly, and their effective use may place greater demands on patients’ digital operational skills (57, 58). For patients with lower DHL, early support in the use of mobile health tools is needed. This includes helping patients learn basic device operation, understand the data, and receive further assistance when difficulties arise.

#### 4.3.4 Interpersonal network level

Patients with higher levels of social support were more likely to belong to the high-level comprehensive DHL profile. This finding is consistent with previous research showing a positive association between DHL and social support among patients with diabetes (59). From the perspective of the HEM, social support is an important factor at the social and community network level and can influence individual health behaviors and health-related capacities through family members, peers, communities, and online social networks. Social support can provide not only emotional encouragement, but also assistance with information interpretation, operational support, and problem-solving resources (60). Qualitative research has further indicated that social support networks are an important channel through which patients with chronic diseases obtain health information. These networks can help filter information, encourage participation, and reduce barriers to information access and understanding (61). In clinical practice, health promotion approaches centered on family, peers, and the community may help improve patients’ DHL through experience sharing, information exchange, and supported use with family members.

#### 4.3.5 Outermost level

From the macro level of the Health Ecological Model, sociocultural environments and macro-level policies are considered key structural drivers of individual health behaviors and capabilities. Since China launched the “Internet Plus Healthcare” initiative in 2018, a series of national policies, including the *Opinions of the General Office of the State Council on Promoting Internet Plus Healthcare* and the *Action Plan for Advancing the “Five-One” Internet Plus Healthcare Services* (2, 3), have been issued to establish a digital health service system covering online consultations, appointment scheduling, mobile payment, electronic health records, chronic disease management, and telemedicine. These macro-level policies provide more accessible and convenient external conditions for chronic disease management. However, the benefits of these policies are not automatically or equally realized, as their effectiveness largely depends on patients’ individual DHL levels. For individuals in the limited DHL profile (C1), a “digital health coaching” model may be implemented, in which family physician teams or diabetes specialist nurses proactively contact patients via telephone, video calls, or instant messaging on a regular basis to help them gradually acquire basic skills such as logging in, completing information forms, and reviewing health reports through guided interpersonal support. For the C2 profile, which is characterized by basic operational skills but weak navigation ability, and the C3 profile, which demonstrates strong operational skills but limited critical appraisal ability, tailored micro-level interventions should be provided, including simplified application interfaces, authoritative information labeling, and decision-support tools, in order to reduce technical barriers to the use of digital health services.

## 5 Limitation

This study has several limitations. First, the sample consisted of young and middle-aged inpatients with diabetes recruited through convenience sampling from two tertiary hospitals in Anhui Province, with fewer younger adults represented in the sample. This recruitment strategy may have introduced selection bias, as hospitalized patients may differ from community or outpatient populations in their digital health needs, disease characteristics, and digital literacy levels. Thus, caution is warranted when generalizing these findings to patients in community settings, outpatient clinics, or primary care institutions. Second, eligibility required the ability to use a smartphone, which may have excluded individuals with limited digital access and, in turn, led to an underestimation of the actual digital divide. Third, the cross-sectional design constrains the ability to infer causality or determine temporal sequences among the observed associations. This design also restricts the assessment of the temporal stability of digital health literacy profiles and may not fully capture dynamic changes in DHL over time. Longitudinal studies are needed to verify the temporal consistency of profiles and strengthen the validity of the observed patterns. Fourth, all key measures were self-reported, reflecting perceived rather than actual digital health literacy. Navigation skills were consistently the lowest across all profiles, which may indicate real difficulties but could also reflect participants’ lack of awareness of their abilities. Future studies should use performance-based tasks (e.g., completing health-related searches on a mobile device) to verify these results.

## 6 Conclusion

This study showed that DHL was not uniform among young and middle-aged adults with diabetes. Four distinct latent profiles were identified: limited DHL, basic application-low navigation, operational strength-limited critical appraisal, and high-level comprehensive DHL. Guided by the HEM, we further found that profile membership was related to multilevel factors, including individual and behavioral characteristics, environmental conditions, and social support. These findings provide empirical support for the stratified assessment of DHL and may inform the development of tailored interventions and health promotion strategies for this population.

## Data Availability

The datasets generated and analyzed during this study are available from the corresponding author upon reasonable request, subject to the execution of a data-sharing agreement to protect participant confidentiality.
