# Supplementary material for: Latent profiles analysis and associated factors of digital health literacy among young and middle-aged adults with diabetes
Source: Front Public Health. 2026 Aug 4;14:1865183. doi: 10.3389/fpubh.2026.1865183 (PMC13481871; doi:10.3389/fpubh.2026.1865183)
Supplement: Supplementary file 2 [file Table_2.docx]

**Supplementary Table 1** Small–Hsiao test for the independence of irrelevant alternatives (IIA) assumption.

| **category** | **χ²** | **df** | ***P*** |
| --- | --- | --- | --- |
| 1 | 9.15 | 14 | 0.821 |
| 2 | 9.39 | 14 | 0.805 |
| 3 | 10.46 | 14 | 0.728 |
| 4 | 13.17 | 14 | 0.514 |

**Supplementary Table 2** Multinomial logistic regression results from sensitivity analysis of factors associated with latent profiles.

| **Variables** | **C1 vs. C4** | | | **C2 vs. C4** | | | **C3 vs. C4** | | |
| --- | --- | --- | --- | --- | --- | --- | --- | --- | --- |
|  | **β** | **OR (95% CI)** | **P** | **β** | **OR (95% CI)** | **P** | **β** | **OR (95% CI)** | **P** |
| Gender (ref: Male) |  |  |  |  |  |  |  |  |  |
| Female | -0.393 | 0.675 (0.285–1.601) | 0.373 | -0.144 | 0.866 (0.297–2.527) | 0.792 | -0.267 | 0.766 (0.216–2.721) | 0.680 |
| Age (ref: 18–37 years) |  |  |  |  |  |  |  |  |  |
| 38-47 years | 0.914 | 2.493 (0.394–15.792) | 0.332 | 0.860 | 2.364 (0.114–49.168) | 0.578 | 0.286 | 1.331 (0.015–118.029) | 0.901 |
| 48‑59 years | 2.321 | 10.187 (1.739–59.678) | 0.010 | 3.072 | 21.590 (1.312–355.225) | 0.032 | 3.886 | 48.710 (0.893–2656.124) | 0.057 |
| Education level (ref: Primary school or below) |  |  |  |  |  |  |  |  |  |
| Junior/Senior high school | -1.533 | 0.216 (0.023–2.061) | 0.183 | -1.790 | 0.167 (0.016–1.692) | 0.13 | -2.962 | 0.052 (0.005–0.555) | 0.014 |
| Bachelor’s degree or above | -2.828 | 0.059 (0.006–0.619) | 0.018 | -2.983 | 0.051 (0.004–0.659) | 0.023 | -5.203 | 0.005 (0.000–0.111) | 0.001 |
| Employment status (ref: Employee) |  |  |  |  |  |  |  |  |  |
| Self-employed | -0.017 | 0.983 (0.414–2.335) | 0.969 | -0.483 | 0.617 (0.176–2.166) | 0.451 | -0.76 | 0.468 (0.099–2.214) | 0.338 |
| Farmer | -1.901 | 0.149 (0.010–2.283) | 0.172 | -1.327 | 0.265 (0.016–4.525) | 0.359 | -1.013 | 0.363 (0.016–8.216) | 0.524 |
| Retired or unemployed | -0.43 | 0.650 (0.266–1.589) | 0.345 | -0.246 | 0.782 (0.250–2.439) | 0.671 | 0.182 | 1.200 (0.284–5.063) | 0.804 |
| Residence (ref: Urban) |  |  |  |  |  |  |  |  |  |
| Rural | 1.452 | 4.272 (0.995–18.346) | 0.051 | 1.633 | 5.119 (0.971–26.978) | 0.054 | 1.399 | 4.050 (0.674–24.326) | 0.126 |
| Income (ref: <5000 RMB) |  |  |  |  |  |  |  |  |  |
| ≥5000 RMB | 0.547 | 1.728 (0.780–3.826) | 0.178 | -0.671 | 0.511 (0.157–1.661) | 0.264 | -0.524 | 0.592 (0.125–2.798) | 0.508 |
| Typr (ref: type 1 |  |  |  |  |  |  |  |  |  |
| type 2 | -1.171 | 0.310 (0.018–5.355) | 0.421 | -0.199 | 0.820 (0.020–33.628) | 0.916 | 0.728 | 2.071 (0.028–152.498) | 0.740 |
| Family history (ref: yes) |  |  |  |  |  |  |  |  |  |
| No | 0.048 | 1.050 (0.551–1.999) | 0.883 | 0.227 | 1.255 (0.532–2.964) | 0.604 | 0.487 | 1.627 (0.576–4.592) | 0.358 |
| Disease duration (ref: <3 years) |  |  |  |  |  |  |  |  |  |
| 3–9 years | 0.285 | 1.330 (0.558–3.174) | 0.520 | -1.108 | 0.330 (0.101–1.082) | 0.067 | 0.233 | 1.262 (0.298–5.354) | 0.752 |
| ≥10 years | 0.277 | 1.319 (0.553–3.146) | 0.533 | -0.46 | 0.631 (0.209–1.905) | 0.414 | -0.052 | 0.950 (0.232–3.893) | 0.943 |
| Complications (ref: 0) |  |  |  |  |  |  |  |  |  |
| 1 | 0.452 | 1.571 (0.683–3.615) | 0.288 | 0.524 | 1.689 (0.558–5.108) | 0.353 | 0.662 | 1.938 (0.521–7.208) | 0.323 |
| ≥2 | 0.92 | 2.509 (0.873–7.212) | 0.088 | 1.455 | 4.282 (1.135–16.161) | 0.032 | 0.765 | 2.148 (0.424–10.890) | 0.356 |
| Comorbid chronic diseases (ref: 0) |  |  |  |  |  |  |  |  |  |
| 1 | -0.595 | 0.551 (0.262–1.162) | 0.117 | 0.255 | 1.291 (0.481–3.463) | 0.612 | 0.022 | 1.023 (0.304–3.437) | 0.971 |
| ≥2 | -0.365 | 0.695 (0.296–1.629) | 0.402 | 0.663 | 1.941 (0.620–6.076) | 0.255 | 1.222 | 3.392 (0.899–12.795) | 0.071 |
| Mobile health tools use (ref: Yes) |  |  |  |  |  |  |  |  |  |
| No | 0.506 | 1.658 (0.662–4.152) | 0.280 | 2.048 | 7.753 (1.121–53.644) | 0.038 | 2.796 | 16.383 (0.918–292.219) | 0.057 |
| Daily internet use (ref: <3 h) |  |  |  |  |  |  |  |  |  |
| 3–5 h | -1.339 | 0.262 (0.127–0.541) | <0.001 | -2.036 | 0.131 (0.047–0.360) | <0.001 | -1.862 | 0.155 (0.044–0.548) | 0.004 |
| >5 h | -1.6 | 0.202 (0.079–0.514) | 0.001 | -2.397 | 0.091 (0.024–0.349) | <0.001 | -3.631 | 0.026 (0.005–0.150) | <0.001 |
| OHIS (ref: <1 time/week or never) |  |  |  |  |  |  |  |  |  |
| Weekly | -1.084 | 0.338 (0.159–0.719) | 0.005 | -2.067 | 0.127 (0.047–0.343) | <0.001 | -2.971 | 0.051 (0.013–0.203) | <0.001 |
| Daily | -1.396 | 0.247 (0.070–0.877) | 0.031 | -4.751 | 0.009 (0.001–0.165) | 0.002 | -4.651 | 0.010 (0.000–0.345) | 0.011 |
| Information source diversity (ref: Single type) |  |  |  |  |  |  |  |  |  |
| ≥2 types | -0.231 | 0.794 (0.349–1.806) | 0.582 | -0.43 | 0.651 (0.243–1.747) | 0.394 | -2.412 | 0.090 (0.027–0.299) | <0.001 |
| Technical anxiety | 0.417 | 1.518 (1.038–2.220) | 0.031 | 1.236 | 3.441 (1.971–6.007) | <0.001 | 1.890 | 6.620 (3.329–13.165) | <0.001 |
| Social support | -0.786 | 0.456 (0.292–0.711) | 0.001 | -1.096 | 0.334 (0.186–0.601) | <0.001 | -1.395 | 0.248 (0.123–0.499) | <0.001 |
| Diabetes Self-Management | -0.154 | 0.857 (0.598–1.226) | 0.398 | -0.481 | 0.618 (0.380–1.005) | 0.052 | -0.458 | 0.633 (0.364–1.101) | 0.105 |
